# 2-(1*H*-Pyrrolo­[2,3-*b*]pyridin-2-yl)pyridine

**DOI:** 10.1107/S1600536812023690

**Published:** 2012-05-31

**Authors:** Ping-Hsin Huang, Yuh-Sheng Wen, Jiun-Yi Shen

**Affiliations:** a Cardinal Tien College of Healthcare & Management, Taipei, Taiwan 231, Republic of China; b Institute of Chemistry, Academia Sinica, Nankang, Taipei, Taiwan, Republic of China; c Department of Chemistry, National Taiwan University, Taipei, Taiwan, Republic of China

## Abstract

In the title compound, C_12_H_9_N_3_, the dihedral angle between the pyridine and aza­indole rings is 6.20 (2)°. In the crystal, pairs of N—H⋯N hydrogen bonds link mol­ecules into inversion dimers.

## Related literature

For the production of luminescent organic/organometallic compounds, see: Liu *et al.* (2000 ▶). For related structures, see: Sakamoto *et al.* (1996 ▶); Huang *et al.* (2011 ▶).
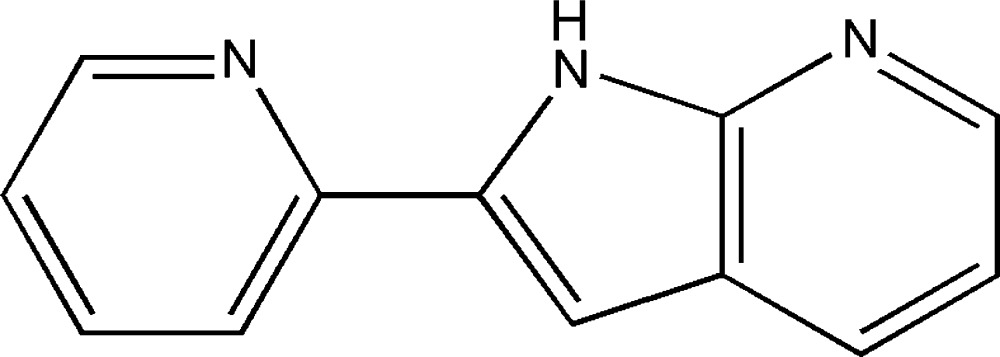


## Experimental

### 

#### Crystal data

C_12_H_9_N_3_
*M* *_r_* = 195.22
Monoclinic, 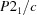

*a* = 10.1416 (10) Å
*b* = 13.7428 (14) Å
*c* = 6.7395 (7) Å
β = 94.331 (2)°
*V* = 936.63 (16) Å^3^
*Z* = 4
Mo *K*α radiation
μ = 0.09 mm^−1^
*T* = 200 K
0.55 × 0.15 × 0.05 mm

#### Data collection

Bruker SMART APEX CCD diffractometer
Absorption correction: multi-scan (*SADABS*; Bruker, 2001 ▶) *T*_min_ = 0.938, *T*_max_ = 0.996
7069 measured reflections
2154 independent reflections
1792 reflections with *I* > 2σa(*I*)
*R*_int_ = 0.056

#### Refinement

*R*[*F* ^2^ > 2σ(*F* ^2^)] = 0.064
*wR*(*F* ^2^) = 0.145
*S* = 1.19
2154 reflections
136 parameters
H-atom parameters constrained
Δρ_max_ = 0.25 e Å^−3^
Δρ_min_ = −0.20 e Å^−3^

### 

Data collection: *SMART* (Bruker, 2001 ▶); cell refinement: *SAINT* (Bruker, 2001 ▶); data reduction: *SAINT*; program(s) used to solve structure: *SHELXS97* (Sheldrick, 2008 ▶); program(s) used to refine structure: *SHELXL97* (Sheldrick, 2008 ▶); molecular graphics: *ORTEP-3* (Farrugia, 1997 ▶); software used to prepare material for publication: *WinGX* (Farrugia, 1999 ▶).

## Supplementary Material

Crystal structure: contains datablock(s) global, I. DOI: 10.1107/S1600536812023690/rk2358sup1.cif

Structure factors: contains datablock(s) I. DOI: 10.1107/S1600536812023690/rk2358Isup2.hkl

Supplementary material file. DOI: 10.1107/S1600536812023690/rk2358Isup3.cml

Additional supplementary materials:  crystallographic information; 3D view; checkCIF report

## Figures and Tables

**Table 1:** Hydrogen-bond geometry (Å, °)

*D*—H⋯*A*	*D*—H	H⋯*A*	*D*⋯*A*	*D*—H⋯*A*
N2—H2⋯N1^i^	0.88	2.10	2.944 (2)	162

Symmetry code: (i) 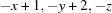.

**Fig. 1.**
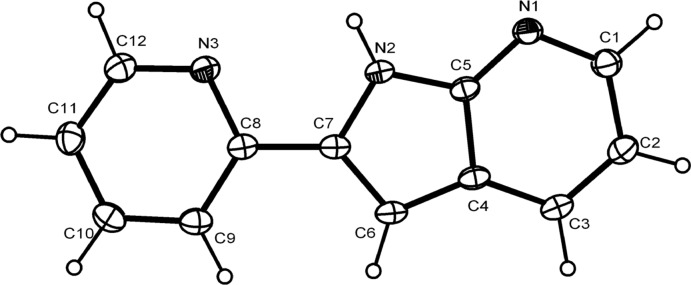
Molecular structure of title compound with the atom numbering scheme. Displacement ellipsoids are drawn at 30% probability level. H atoms are shown as small spheres of the arbitrary radii.

## supplementary crystallographic information

### Comment

The title compound, has been shown to be an precursor for the production of luminescent organic/organometallic compound (Liu *et al.*, 2000). A one pot synthesis of a 7-azaindole substituted in the 2-position has been achieved by Pd complex catalyzed Cross-coupling reaction of 2-bromopyridine (Sakamoto *et al.*, 1996), in high yield (see Scheme). The molecular structure is shown in Fig. 1. The dihedral angle between the pyridine and azaindole rings is 6.20 (2)°, and that between the pyridine and pyrrole rings at 7-azaindole is 0.35 (2)° (Huang *et al.*, 2011). Weak intermolecular N2—H2···N1^i^ (see Table 1) interactions help to stabilize the crystal structure - formation of centrosymmetrical dimers. Symmetry code: (i) -*x*+1, -*y*+2, -*z*.

### Experimental

The compound was synthesized by the following procedure (Liu *et al.*, 2000); (Sakamoto *et al.*, 1996). A solution of 1-(benzenesulfonyl)-2-(2-pyridyl)-7-azaindole (2.00 g, 5.97 mmol), ethanol (340 ml), and 10% aqueous NaOH (34 ml) was heated at reflux overnight. The resulting mixture was concertrated, and the residue was dissolved in CH_2_Cl_2_. The organic solution was washed with water and aqueous Na_2_CO_3_, dried, and concertrated. The residue was purified by column chromatography using CH_2_Cl_2_/CH_3_OH (20:1) as eluent, followed by recrystallization from CH_2_Cl_2_ and hexane to yield 0.82 g (70%) of title compound as a white solid. Crystals suitable for X-ray diffraction were grown from a CH_2_Cl_2_ solution layered with hexane at room temperature. ^1^H NMR (CDCl_3_): 10.90 (br s, 1H), 8.69 (ddd, 1H, J = 4.8, 1.6, 1.0 Hz), 8.46 (dd, 1H, J = 4.8, 1.6 Hz), 7.98 (dd, 1H, J = 7.8, 1.3 Hz), 7.85 (m, 1H), 7.76 (td, 1H, J = 7.8, 1.7 Hz), 7.24 (ddd, 1H, J = 7.8, 4.8, 1.2 Hz), 7.12 (dd, 1H, J = 4.8, 1.8 Hz), 6.99 (d, 1H, J = 1.8 Hz). Anal. Calcd for C_12_H_9_N_3_: C, 73.83; H, 4.65; N, 21.52. Found: C, 73.26; H, 4.48; N, 21.58.

### Refinement

H atoms were located geometrically and treated as riding atoms, with C—H = 0.93Å, and with *U*_iso_(H) = 1.2*U*_eq_(C).

### Crystal data

**Table:**

C_12_H_9_N_3_	*F*(000) = 408
*M**_r_* = 195.22	*D*_x_ = 1.384 Mg m^−^^3^*D*_m_ = 1.384 Mg m^−^^3^*D*_m_ measured by not measured
Monoclinic, *P*2_1_/*c*	Mo *K*α radiation, λ = 0.71073 Å
Hall symbol: -P 2ybc	Cell parameters from 1871 reflections
*a* = 10.1416 (10) Å	θ = 2.5–26.3°
*b* = 13.7428 (14) Å	µ = 0.09 mm^−^^1^
*c* = 6.7395 (7) Å	*T* = 200 K
β = 94.331 (2)°	Needle, colourless
*V* = 936.63 (16) Å^3^	0.55 × 0.15 × 0.05 mm
*Z* = 4	

### Data collection

**Table:**

Bruker SMART APEX CCD diffractometer	2154 independent reflections
Radiation source: fine-focus sealed tube	1792 reflections with *I* > 2σa(*I*)
Graphite monochromator	*R*_int_ = 0.056
ω scans	θ_max_ = 27.5°, θ_min_ = 2.0°
Absorption correction: multi-scan (*SADABS*; Bruker, 2001)	*h* = −13→13
*T*_min_ = 0.938, *T*_max_ = 0.996	*k* = −17→17
7069 measured reflections	*l* = −8→8

### Refinement

**Table:**

Refinement on *F*^2^	Primary atom site location: structure-invariant direct methods
Least-squares matrix: full	Secondary atom site location: difference Fourier map
*R*[*F*^2^ > 2σ(*F*^2^)] = 0.064	Hydrogen site location: inferred from neighbouring sites
*wR*(*F*^2^) = 0.145	H-atom parameters constrained
*S* = 1.19	*w* = 1/[σ^2^(*F*_o_^2^) + (0.0366*P*)^2^ + 0.2976*P*] where *P* = (*F*_o_^2^ + 2*F*_c_^2^)/3
2154 reflections	(Δ/σ)_max_ < 0.001
136 parameters	Δρ_max_ = 0.25 e Å^−^^3^
0 restraints	Δρ_min_ = −0.20 e Å^−^^3^

### Special details

**Table:**

Geometry. All s.u.'s (except the s.u. in the dihedral angle between two l.s. planes) are estimated using the full covariance matrix. The cell s.u.'s are taken into account individually in the estimation of s.u.'s in distances, angles and torsion angles; correlations between s.u.'s in cell parameters are only used when they are defined by crystal symmetry. An approximate (isotropic) treatment of cell s.u.'s is used for estimating s.u.'s involving l.s. planes.
Refinement. Refinement of *F*^2^ against ALL reflections. The weighted *R*-factor *wR* and goodness of fit *S* are based on *F*^2^, conventional *R*-factors *R* are based on *F*, with *F* set to zero for negative *F*^2^. The threshold expression of *F*^2^ > σ(*F*^2^) is used only for calculating *R*-factors(gt) *etc*. and is not relevant to the choice of reflections for refinement. *R*-factors based on *F*^2^ are statistically about twice as large as those based on *F*, and *R*-factors based on ALL data will be even larger.

### Fractional atomic coordinates and isotropic or equivalent isotropic displacement parameters (Å^2^)

**Table:**

	*x*	*y*	*z*	*U*_iso_*/*U*_eq_	
N1	0.66995 (16)	0.94501 (12)	0.0034 (2)	0.0382 (4)	
N2	0.50593 (15)	0.92225 (11)	0.2357 (2)	0.0324 (4)	
H2	0.4394	0.9513	0.1684	0.039*	
N3	0.27130 (16)	0.91454 (12)	0.4136 (2)	0.0371 (4)	
C1	0.7961 (2)	0.92562 (16)	−0.0253 (3)	0.0418 (5)	
H1	0.8296	0.9475	−0.1454	0.050*	
C2	0.8818 (2)	0.87517 (16)	0.1101 (3)	0.0412 (5)	
H2A	0.9707	0.8639	0.0808	0.049*	
C3	0.8377 (2)	0.84179 (14)	0.2859 (3)	0.0391 (5)	
H3	0.8948	0.8070	0.3790	0.047*	
C4	0.70684 (19)	0.86035 (13)	0.3238 (3)	0.0333 (4)	
C5	0.62979 (18)	0.91210 (13)	0.1741 (3)	0.0316 (4)	
C6	0.62305 (19)	0.84091 (13)	0.4777 (3)	0.0351 (5)	
H6	0.6466	0.8073	0.5984	0.042*	
C7	0.50115 (19)	0.87982 (13)	0.4198 (3)	0.0323 (4)	
C8	0.37880 (18)	0.87873 (13)	0.5192 (3)	0.0327 (4)	
C9	0.3725 (2)	0.84151 (14)	0.7108 (3)	0.0411 (5)	
H9	0.4501	0.8179	0.7828	0.049*	
C10	0.2536 (2)	0.83931 (15)	0.7945 (3)	0.0465 (6)	
H10	0.2474	0.8138	0.9244	0.056*	
C11	0.1429 (2)	0.87497 (16)	0.6860 (3)	0.0460 (5)	
H11	0.0586	0.8738	0.7388	0.055*	
C12	0.1579 (2)	0.91213 (16)	0.5003 (3)	0.0440 (5)	
H12	0.0817	0.9380	0.4283	0.053*	

### Atomic displacement parameters (Å^2^)

**Table:**

	*U*^11^	*U*^22^	*U*^33^	*U*^12^	*U*^13^	*U*^23^
N1	0.0389 (9)	0.0415 (9)	0.0336 (9)	0.0069 (7)	−0.0001 (7)	0.0021 (7)
N2	0.0326 (8)	0.0329 (8)	0.0306 (8)	0.0031 (6)	−0.0059 (6)	0.0030 (7)
N3	0.0347 (9)	0.0376 (9)	0.0382 (9)	−0.0009 (7)	−0.0026 (7)	0.0040 (7)
C1	0.0422 (11)	0.0459 (12)	0.0373 (11)	0.0069 (9)	0.0026 (9)	0.0005 (9)
C2	0.0352 (11)	0.0435 (12)	0.0444 (12)	0.0067 (9)	−0.0013 (9)	−0.0031 (10)
C3	0.0366 (11)	0.0361 (11)	0.0426 (12)	0.0037 (8)	−0.0104 (9)	0.0006 (9)
C4	0.0379 (10)	0.0259 (9)	0.0341 (10)	−0.0004 (8)	−0.0101 (8)	−0.0009 (8)
C5	0.0336 (10)	0.0281 (9)	0.0319 (10)	0.0027 (8)	−0.0050 (8)	−0.0030 (8)
C6	0.0390 (11)	0.0298 (10)	0.0344 (10)	−0.0013 (8)	−0.0106 (8)	0.0053 (8)
C7	0.0382 (10)	0.0253 (9)	0.0321 (10)	−0.0037 (8)	−0.0065 (8)	0.0015 (8)
C8	0.0357 (10)	0.0253 (9)	0.0358 (10)	−0.0038 (7)	−0.0054 (8)	−0.0002 (8)
C9	0.0453 (12)	0.0367 (11)	0.0401 (11)	−0.0023 (9)	−0.0050 (9)	0.0062 (9)
C10	0.0566 (14)	0.0433 (12)	0.0397 (12)	−0.0035 (10)	0.0054 (10)	0.0083 (10)
C11	0.0410 (12)	0.0458 (12)	0.0521 (13)	−0.0032 (10)	0.0094 (10)	0.0023 (10)
C12	0.0393 (11)	0.0444 (12)	0.0473 (12)	0.0000 (9)	−0.0027 (9)	0.0011 (10)

### Geometric parameters (Å, º)

**Table:**

N1—C5	1.328 (2)	C4—C6	1.415 (3)
N1—C1	1.335 (3)	C4—C5	1.420 (3)
N2—C5	1.360 (2)	C6—C7	1.376 (3)
N2—C7	1.376 (2)	C6—H6	0.9500
N2—H2	0.8800	C7—C8	1.454 (3)
N3—C12	1.329 (3)	C8—C9	1.395 (3)
N3—C8	1.349 (2)	C9—C10	1.369 (3)
C1—C2	1.396 (3)	C9—H9	0.9500
C1—H1	0.9500	C10—C11	1.383 (3)
C2—C3	1.377 (3)	C10—H10	0.9500
C2—H2A	0.9500	C11—C12	1.371 (3)
C3—C4	1.393 (3)	C11—H11	0.9500
C3—H3	0.9500	C12—H12	0.9500
			
C5—N1—C1	114.66 (18)	C7—C6—H6	126.4
C5—N2—C7	109.17 (15)	C4—C6—H6	126.4
C5—N2—H2	125.4	N2—C7—C6	109.17 (17)
C7—N2—H2	125.4	N2—C7—C8	120.62 (16)
C12—N3—C8	116.82 (17)	C6—C7—C8	130.19 (18)
N1—C1—C2	124.1 (2)	N3—C8—C9	122.01 (18)
N1—C1—H1	117.9	N3—C8—C7	115.95 (17)
C2—C1—H1	117.9	C9—C8—C7	122.04 (18)
C3—C2—C1	120.04 (19)	C10—C9—C8	119.5 (2)
C3—C2—H2A	120.0	C10—C9—H9	120.2
C1—C2—H2A	120.0	C8—C9—H9	120.2
C2—C3—C4	118.22 (19)	C9—C10—C11	118.7 (2)
C2—C3—H3	120.9	C9—C10—H10	120.7
C4—C3—H3	120.9	C11—C10—H10	120.7
C3—C4—C6	137.09 (19)	C12—C11—C10	118.2 (2)
C3—C4—C5	116.24 (18)	C12—C11—H11	120.9
C6—C4—C5	106.67 (17)	C10—C11—H11	120.9
N1—C5—N2	125.47 (17)	N3—C12—C11	124.7 (2)
N1—C5—C4	126.71 (18)	N3—C12—H12	117.6
N2—C5—C4	107.81 (17)	C11—C12—H12	117.6
C7—C6—C4	107.17 (17)		

### Hydrogen-bond geometry (Å, º)

**Table:**

*D*—H···*A*	*D*—H	H···*A*	*D*···*A*	*D*—H···*A*
N2—H2···N1^i^	0.88	2.10	2.944 (2)	162

Symmetry code: (i) −*x*+1, −*y*+2, −*z*.

## References

[bb1] Bruker (2001). *SMART*, *SAINT* and *SADABS* Bruker AXS Inc., Madison, Wisconsin, USA.

[bb2] Farrugia, L. J. (1997). *J. Appl. Cryst.* **30**, 565.

[bb3] Farrugia, L. J. (1999). *J. Appl. Cryst.* **32**, 837–838.

[bb4] Huang, P.-H., Lin, K.-L. & Wen, Y.-S. (2011). *Acta Cryst.* E**67**, o109.

[bb5] Liu, S. F., Wu, Q., Schmider, H. L., Aziz, H., Hu, N. X., Popovic, Z. & Wang, S. (2000). *J. Am. Chem. Soc.* **122**, 3671–3678.

[bb6] Sakamoto, T., Kondo, Y., Takazawa, N. & Yamanaka, H. (1996). *J. Chem. Soc. Perkin Trans. 1*, pp. 1927–1934.

[bb7] Sheldrick, G. M. (2008). *Acta Cryst.* A**64**, 112–122. 10.1107/S0108767307043930 18156677

